# Deciphering the gut microbiome’s metabolic code: pathways to bone health and novel therapeutic avenues

**DOI:** 10.3389/fendo.2025.1553655

**Published:** 2025-05-22

**Authors:** Daniel Hwang, Esther Chong, Yanxiu Li, Yanling Li, Kangsan Roh

**Affiliations:** ^1^ Oxford Academy, Cypress, CA, United States; ^2^ The Science Academy STEM Magnet., North Hollywood, CA, United States; ^3^ Crean Lutheran High School, Irvine, CA, United States; ^4^ Cardiovascular Research Center, Massachusetts General Hospital, Harvard Medical School, Boston, MA, United States

**Keywords:** gut microbiome, bone health, osteoporosis, microbial metabolites, bone remodeling

## Abstract

The gut microbiome plays an important role in the protection against various systemic diseases. Its metabolic products profoundly influence a wide range of pathophysiological events, including the regulation of bone health. This review discusses the recently established connections between the gut microbiome and bone metabolism, focusing on the impact of microbiome-derived metabolites such as SCFAs, Bile Acids, and tryptophan to the control of bone remodeling and immunoreactions. Recent advances in metagenomics and microbiome profiling have unveiled new exciting therapeutic opportunities, ranging from the use of probiotics, prebiotics, engineered microbes, and to fecal microbiota transplantation. Understanding of the interplay among diet, microbiota, and bone health provides new avenues for tailored interventions aimed at reducing disease risk in osteoporosis and other related disorders. By drawing knowledge from microbiology, metabolism, and bone biology, this review highlights the potential of microbiome-targeted therapies to transform skeletal health and the management of bone diseases.

## 1 Introduction

Trillions of microorganisms live in the human gut, and they form a complex but highly balanced ecosystem (1). Accumulating evidence demonstrates that disturbing this complicated ecosystem profoundly affects bone density and may influence the risk of osteoporosis (2, 3). Dysbiosis or gut microbiota imbalance has been strongly associated as the cause of increased bone resorption and reduced bone formation through immune modulation, inflammation, and impaired nutrient absorption. Menopause and aging are particularly to blame for disturbances of microbiota, triggering inflammatory pathways that increase osteoclast activity and bone loss (4–6). Emerging research also links gut microbiota with inflammation that can affect conditions like rheumatoid arthritis (7). Various gut microbiome metabolic products such as short-chain fatty acids (SCFAs), bile acids, and tryptophan metabolites significantly influence bone metabolism and health (8, 9). SCFAs, including acetate, propionate, and butyrate, directly inhibit osteoclastogenesis and enhance osteoblast function, and hence maintain bone structure and bone density. SCFAs facilitate the absorption of calcium by lowering intestinal pH, enhancing calcium solubility, and providing a mechanistic link between diet, microbiome metabolism, and bone mineralization. Recent advances in metagenomics and functional profiling introduce new concepts toward how the gut microbiome may systemically influence overall human health (10). For instance, metagenomics analysis found some microbial gene clusters that are responsible for the production of health-beneficial metabolites including SCFAs, and this identified targeted possibilities of microbiome therapies in osteoporosis (9, 11). Metagenomic sequencing, functional metagenomics, and phylogenetic profiling provide a better understanding of how the gut microbiome regulates bone metabolism through diet, supplementation, or designed microbial therapies. In this review, we discuss various microbiome-targeted interventions such as probiotics, prebiotics, engineered microbes, and fecal microbial transplantation (FMT) in the aspects of bone health. Preclinical and clinical studies affirm the efficacy of these interventions to restore microbiome balance, reduce inflammation, and promote mineral absorption, all of which highlight their therapeutic potential against osteoporosis (12, 13). We aim to bring together different perspectives from the disciplines of microbiology, metabolism, and bone biology with regard to gut microbiome manipulation; it may well translate into new opportunities to optimally manage skeletal health-related diseases (7, 10).

## 2 Unraveling the gut microbiome

The human gut microbiome consists of hundreds of trillions of microorganisms and plays a crucial role in maintaining human health by supporting digestion, immune function, metabolic balance, and more (14). Disruptions in this huge and intricate microbial network (dysbiosis) are associated with a variety of health conditions, including inflammatory bowel disease (IBD), obesity, diabetes, cardiovascular disease, neurological conditions like Alzheimer’s disease, and autoimmune conditions like rheumatoid arthritis and systemic lupus erythematosus (15–17). Microbiome-focused dietary interventions supplemented with fermented foods significantly increased microbial diversity and reduced markers of inflammation. For instance, a 10-week study demonstrated that subjects who consumed fermented foods experienced notable increases in gut microbiota diversity accompanied by significant reductions in 19 inflammatory cytokines, including IL-6, IL-10, and IL-12b (18). Metagenomic DNA sequencing technologies have been developed to study the diversity of gut microbes (19). Metagenomic sequencing refers to a methodology used to examine genetic material derived directly from microbial populations, enabling researchers to identify microbial genes and their biochemical activities (20, 21). A study showed the alternative ways, through which the gut microbiome has been influencing glycan, amino acid, and xenobiotics metabolisms; such metabolism usually forms a pattern of enrichment in Clusters of Orthologous Groups (22). Other studies applied 16S rRNA gene sequencing to understand how a gut microbiome could promote immune response and absorb food for the liberation of nutrition to the human host (23). However, differences in the efficiency of DNA extraction, sequencing depth, and sample handling can lead to biases with the potential to cause significant errors in the estimation of microbial abundance (24). Separately, simulation studies support this approach because gene functional prediction indicates specific pathways, providing information on the connective chains of reactions expected to occur for gene activity. Because phylogenetic profiling defines gene-gene co-occurrence patterns across species, this method can be used to see in what ways or where the absence or presence of a gene may affect metabolic capabilities in prokaryotes (25). It shows the importance of gene co-occurrence within a microbial network where the interactions go beyond digestion and relate to phenotypic traits (24). One can apply insights from these technologies to work towards therapeutic interventions and diets by the health-promoting properties of the gut microbiome (26). These microbiome-targeted interventions show promising preclinical efficacy and emerging clinical potential in treating neurological disorders such as Alzheimer’s disease, Parkinson’s disease, stroke, and epilepsy by modulating microbial composition and metabolism, thereby influencing disease pathology (27).

The composition of the gut microbiome can predict the susceptibility of a host to some diseases (28). The microbiome uses colonization resistance to prevent infection in the host, where the commensal microbiota of the microbiome competes against invading microorganisms (29, 30). The gut microbiome is known to act to modulate immune homeostasis and serve as a barrier from several diseases at this gut-symbiosis interface (17). SCFAs represent the major products of the gut microbiome and play an essential role in gut homeostasis (30). Butyrate controls the adaptive and innate immune cells, while SCFAs protect against dysbiosis and maintain gut health (29). Finally, antigens are presented through antigen-presenting cells to T cells and afterward may bias the T cells into Th1, Th2, or Th17 cells (17). This interaction downregulates gut macrophage activity and reduces inflammation through modulation of T cell responses in the gut (30). The metabolic activity of the gut microbiota regulates diseases beyond the gut barrier and also serves as an indicator of the local immune system (31). While the activation of the pattern-recognition receptors in the gut controls the appropriate immune response, the barrier function is a multi-step system of the gut that reduces the adhesion of bacteria and regulates nutrition absorption (30). A balanced gut microbiota notably regulates immune mediators in the GIT and profoundly strengthens gut barrier function (29). In fact, studies in mice have shown that proper mucus formation depends on an actively growing, diversely structured microbiota (32). The host gut microbiota regulates the secretion of mucosal digestive enzymes, such as meprin-β, into the small intestine to modulate mucus production (32). Furthermore, on a diet devoid of dietary fibers, the mucus layer is degraded when the bacteria-degrading mucus thrives. On the other hand, leaky gut has recently been associated with pathogenesis leading to several inflammatory ailments (33). It is observed that a healthy gut mucus layer and proper immune system depend on the proper interaction ability of the gut microbes within their host (30). It is, therefore, important to understand the complexity, composition, regulation, and functioning of gut microbiota in order to achieve therapeutic breakthroughs and develop the best microbiome-specific therapies that would promote human health and prevent disease (17).

The diversity of the microbial community in the gut is a direct result of the biochemical profile of food that the host uptakes (34). Dysbiosis is caused by an unstable microbiome leading to changes in composition and diversity of the gut microbiome, which has been associated with a series of circumstances, including the loss of balanced nutrition owing to high sugar and/or low fiber, which leads to disruption to the host’s metabolism. In addition, antibiotic overuse and the consequential antimicrobial resistance of gut bacteria also cause dysbiosis (35, 36). This pathological situation often results in an overgrowth of opportunistic pathogenic microorganisms in conjunction with the loss of beneficial microorganisms (35). Indeed, therapeutic strategies supporting microbiome stability to restore its diversity have been found to play key roles in maintaining proper immune function and long-term health. This form of maintenance requires tailored therapeutic strategies and includes a range of factors, including prebiotics and probiotics, as well as changes in diet that foster microbial diversity and overall health (37, 38). Importantly, currently available microbiome-targeted therapies provide innovative means to suppress dysbiosis and its role in numerous inflammatory diseases (39, 40). Together, understanding the microbiome and its interactions with diverse ranges of different hosts is essential to prevent or intervene in dysbiosis (41).

## 3 Bone metabolism: foundations and failures

Bone remodeling is a constantly ongoing critical homeostatic process in the body throughout our lives (42). It is tightly regulated by the precise continual resorption of the bone by the osteoclasts and its counteracting, continual formation by the osteoblasts (43, 44). The receptor activator of nuclear factor (RANK) and its ligand, RANKL, are crucial in the promotion of bone resorption by osteoclasts (44). In addition, the balance between estrogen and testosterone properly controls osteoclast activity, promoting bone formation to help maintain bone mass, and also protect against osteoporosis, most notably in postmenopausal women (45). This suggests that an increase in osteoclast activity can be caused by parathyroid hormone (PTH), which acts on osteoblasts and influences the RANKL/Osteoprotegerin (OPG) signaling system (46). Mechanically, following receptor binding of PTH, a signal transduction cascade is initiated and alters the RANKL/OPG-producing profile - with an increase in the RANKL profile and a decrease in the OPG profile (47). This leads to the overall net effect that would favor osteoclast development and productivity levels. On the other hand, calcitonin inhibits osteoclast activity by binding to its cognitive receptors on osteoclasts and triggers the signaling pathways that reduce their bone-resorbing ability. Moreover, hormonal balancing mechanisms would render control over the activity of osteoblasts and osteoclasts and properly maintain the innate homeostasis in the processes essential for the formation and function of bone (43). In the absence of such balance, bone loss will accelerate and predispose to fracture (44). On the other hand, overactivation of osteoblasts may lead to the overproduction of bone and cause abnormalities in bones themselves and marrows therein (48).

Several bone resistance training programs reported the ability to improve bone mineral density (BMD) in postmenopausal women with low bone mass (49–51). The interest in preventive strategies to build bone mass and demand for adequate assessment tools to properly calculate the risk for fractures have grown dramatically in recent years (52). Accurately predicting bone health allows for tailored therapies to address specific weaknesses, improving osteoporosis management and reducing fracture risk (53, 54). Trabeculae, the spongy part of bone, forms thin plates and rod-like structures, enabling it to perform its function of bearing weight and resisting forces more appropriately (55). Also, minerals in the matrix of a healthy bone affect the strength of the bone, while collagen fibers are arranged and cross-linked for the flexibility or bending ability of the bone. Advanced imaging techniques like QCT, High-resolution peripheral computed tomography (HR-pQCT), Trabecular Bone Score (TBS), and Finite Element Analysis (FEA) provide precise and immediate determination of multiple bone strength parameters, such as cortical bone density, cortical thickness, cortical porosity, and density (56). Spectroscopic tools like Fourier Transform Infrared (FTIR) spectroscopy and Raman spectroscopy provide a deeper understanding of the composition of bone with respect to mineral and collagen properties than can be obtained with standard BMD testing (57). These imaging techniques enhance the diagnosis of and guide treatment strategies (58). However, despite these advancements, metabolic failures remain the primary challenge in bone health management.

Bone metabolic failures underlie bone conditions. Rheumatoid arthritis (RA) and osteoarthritis (OA) point to the role of imbalanced metabolism (59). The inflammatory cytokines in RA cause disruption in the balance between RANKL and OPG, triggering bone resorption with enhanced fracture risk (60). In contrast, in OA, there is cartilage degradation and bone spur development, illustrating how mechanical stress and abrasive wear weaken bone (61). Also, hormonal imbalances with reductions in estrogen enhance osteoclast-mediated activity, leading to postmenopausal osteoporosis in women (62). The treatment of these metabolic failures remains paramount despite the availability of advanced diagnostic tools since viable interventions are limited with these advanced diagnostic methods.

In RA, several cytokines have been identified to disrupt the balance between RANKL, an activator of osteoclast activity, and its inhibitor OPG, resulting in excessive bone resorption (63). Despite the recent improvements in treatment, complete recovery of damaged bones in RA remains unlikely, considering that inflammatory cytokines persistently inhibit new bone formation (63). However, in the case of patients with OA, cartilage wears down slowly and results in a loss of elasticity and characteristics as a shock absorber. As the cartilage degrades, it exposes the bone below and results in the formation of bone spurs (64). A study outlined that osteoarthritis is an outcome of physical trauma and day-to-day use and wear of the joints (65). In comparison, the autoimmune response characterizing RA tends to damage the bones. In this process, immune cells invade the tissue membrane that triggers an inflammatory response in the synovium and cause erosion of the neighboring bones.

Numerous genetic factors, including Vitamin D receptor gene polymorphisms, are known to interact with lifestyle choices to affect bone health. Specific polymorphisms of genes of the Vitamin D receptor, such as haplotype A-T-G for rs7975232, rs1544410, and rs73731236, have been associated with low BMD, increased susceptibility to osteoporosis, and other bone diseases (66). Indeed, Vitamin D is a crucial nutrient and promotes calcium absorption, and adequate Vitamin D levels help build strong bones and reduce osteoporosis risk (67). Exercise also reduces bone loss in older age. Regular activities stimulate the formation of bones and likely reduce bone fractures with a significantly diminished chance of developing osteoporosis (68). Smoking negatively affects the flow of hormones and impairs calcium absorption and bone metabolism (69). This leads to low bone mass and density and contributes to a high fracture risk (69). On the other hand, sex hormones, important for osteoblast activation, are negatively affected by smoking. Moderate alcohol consumption has a complex impact on BMD via direct effects on bone mineral metabolism. Moderate alcohol consumption may have varying effects on bone density, especially in postmenopausal women, although its impact on estrogen levels remains complex. Acute alcohol intoxication may lead to the development of transient hypoparathyroidism with consequent hypocalcemia, whereas chronic alcoholism reduces levels of vitamin D metabolites and impairs calcium absorption. Alcohol blunts osteoblast activity, as low levels of osteocalcin, a protein involved in new bone formation, are observed in alcoholics (69). Some influences and risk factors important in determining the state of health of our bones are genetically predetermined - phenomena such as peak bone mass; most of the others will be determined by the accumulation of lifestyle choices that we make over a lifetime (68). Maintaining strong bone structure is essential for mobility, independence, and overall well-being.

Most pathological conditions affecting the bone can benefit from a comprehensive approach to treatment. Traditional treatment methods include bisphosphonates, hormone replacement therapy (HRT), and the supplementation of calcium and vitamin D. Among the newer therapeutic options for osteoporosis are teriparatide and romosozumab. Vitamin D deficiency can delay fracture healing even when surgical procedures are correctly performed (67). Bisphosphonates, including alendronate and zoledronic acid, are used to deal with osteoporosis. Binding to bone minerals inhibits osteoclast activity and thus limits bone resorption. At menopause, reduced estrogen levels disrupt the balance of bone remodeling, which leads to increased bone resorption followed by bone loss. HRT restores estrogen levels, suppresses osteoclast activity, maintains osteoblast function, and thus preserves bone mass in postmenopausal women (70). However, although HRT helps retain bone density, its widespread usage has been limited by its non-trivial adverse effects, such as the occurrence of breast cancer and the potential for stroke and clot formation. Thus, HRT has been very cautiously prescribed to clearly specified patterns of patients in well-controlled medical settings (71). New therapeutic possibilities include romosozumab and anabolic agents such as teriparatide. Romosozumab is a monoclonal antibody that targets a key pathway involved in bone formation. A reduction in the risk of fragility fractures was considerably noted in the romosozumab group compared to that in the control patient population with increased BMD at the lumbar spine, total hip, and femoral neck among the skeletal sites (72). On the other hand, teriparatide is a synthetic form of parathyroid hormone that stimulates new bone formation by enhancing osteoblast activity, resulting in increased BMD and reduced fracture risk (73).

## 4 Metabolic intersections: microbiome and bone health

SCFAs inhibit osteoclast activity and bone resorption and, thus, indirectly prevent bone loss. Mechanistically, they can also activate GPR41 and GPR43 receptors on osteoclast precursors, preventing their differentiation into mature bone-resorbing osteoclasts (74). In a mouse study, gut dysbiosis lowered butyrate levels. FMT upregulated GPR43 through the elevated levels of butyrate in the bone tissue, leading to the suppression of the osteoclast genesis process and heightened osteoblast function, thus reducing excessive resorption (74). On the other hand, healthy bone promotion by SCFAs is governed through the activation of Tregs. Tregs block the excessive induction of inflammatory responses while polarizing macrophages into an anti-inflammatory M2 phenotype. SCFAs induce M2 macrophage anti-inflammatory polarization through butyrate-mediated STAT6 activation. They also promote regulatory T-cell differentiation, reduce exaggerated inflammatory responses, and support the maintenance of bone mass (75, 76). Fiber-rich diets help prevent bone loss and maintain balanced bone renewal (36, 77–79).

The gut microbiota converts primary bile acids into secondary forms through various enzymatic processes in the intestines. And bile acid signaling is known to play a role in bone metabolism through receptors such as FXR and TGR5 (80). The interaction between blood concentrations of bile salts and bone density suggests that bile acid signaling directly affects bone metabolism. Dietary fibers enhance the growth of beneficial gut microbiota and influence bile acid metabolism, leading to altered bile acid pools. These changes in the bile acid pool may influence bone health by affecting receptor activation and calcium homeostasis. While TGR5 and vitamin D receptor activation could play a role, regulating the gut microbiota through FMT or probiotics may provide therapeutic potential for diseases related to dysregulated bile acid metabolism (81, 82).

The kynurenine pathway metabolizes the major part of total tryptophan into kynurenine in the liver through induction of the rate-limiting enzyme indoleamine-2,3-dioxygenase or, to a lesser extent, by induction of tryptophan 2,3-dioxygenase in other tissues (83). Likewise, the gut microbiota metabolizes tryptophan into various indoles, such as indole-3-acetic acid and indole-3-aldehyde, which might be the ligands of the aryl hydrocarbon receptor pathway in mediating bone health and homeostasis (84). Indole is a potent AhR ligand that influences multiple metabolic pathways relevant to bone health. It translocates into the nucleus, where AhR heterodimerizes with the ARNT protein and binds to the target gene promoter at XRE, and initiates the transcription of XRE. Indole compounds such as indole-3-aldehyde and indole-3-acetic acid increase bone mass by promoting osteoblast differentiation and reducing bone resorption via the AhR pathway (85). In addition, indoles activate microbiota-derived AhR and promote intestinal health by regulating immune responses and epithelial barrier function. AhR activation by microbial indoles reduces the expression of pro-inflammatory cytokines, modulates inflammation-mediated bone resorption and also supports bone health by regulating osteoclast activity (86). A study showed that gut microbiota metabolism of tryptophan-induced AhR signaling acted as a master switch for renal fibrosis (87). This suggested that microbial tryptophan metabolites modulate renal inflammation and fibrosis through the AhR pathway. Moreover, high kynurenine levels promote osteoclastogenesis by increasing the RANKL expression, which is considered to be the major cytokine that induces osteoclast differentiation (86). Therefore, kynurenine resorbs bone and enhances its resorption. However, kynurenine was also known to negatively modulate osteoblast activity and bone formation by suppressing anabolic pathways. Kynurenine induces RANKL expression and promotes osteoclast differentiation while inhibiting osteoblast activity. The imbalance will shift the normal course to age-related osteoporosis and increase the risk of fractures (86). Products of tryptophan metabolism by gut microbiome induce the AhR and pregnane X receptor pathways. This allows them to regulate gut barrier homeostasis and immune modulation (88). Gut microbiota helps maintain gut barrier integrity and regulates inflammatory responses through the metabolite activators of AhR. Indeed, activation of AhR by some indole derivatives showed neuroprotective and anti-inflammatory effects (89). Together, gut microbiota metabolizes tryptophan into various active compounds, including indoles and kynurenine, which play a key role in bone health. **Table 1** summarizes the effects of key microbiome-derived metabolites on bone health.

**Table 1:** Microbiome-derived metabolites and their effects on bone health (Section 3).

Metabolite	Source	Mechanism of action	Impact on bone health	References
Short-chain fatty acids (SCFAs)	Gut bacteria fermentation of dietary fibers (*Lactobacillus*, *Bifidobacterium*)	Modulate immune responses and stimulate production of regulatory T cells (Tregs)	Support bone growth, inhibit resorption of bone, improve bone mineral density (BMD)	(76, 131, 132)
Bile acids (BAs)	Modified by *Clostridium* and *Bacteroides* in the liver	Activate FXR and TGR5 signaling cascades and modulate calcium absorption and homeostasis	Regulate bone remodeling, enable mineralization, stimulate osteoblast	(133–135)
Tryptophan metabolites	Gut microbiota degradation (*Clostridium*, *Bacteroides*)	Activate AhR pathway, modulate immunity and maintain epithelial barrier integrity	Increase osteoblast differentiation, inhibit bone resorption, suppress	(89, 136–138)
Butyrate	SCFA derived from fiber fermentation	Activated STAT6 polarizes anti-inflammatory M2 phenotype of macrophages	Causes inhibition of osteoclastogenesis, increases bone formation	(76, 95, 139)
Indoles	Secondary tryptophan catabolic products	Activate AhR and inhibit production of pro-inflammatory cytokines	Reduce bone resorption and add bone mass	(86, 140)
Phenolic compounds	Fermentation of polyphenols by gut bacteria (*Lactobacillus*)	Regulate oxidative stress and inflammation	Protect against bone resorption by inhibiting oxidation damage	(141)
Hydrogen Sulfide (H2S)	Breakdown of sulfur amino acids by sulphate-reducing bacteria	Strictly controls bone resorption and osteoclast differentiation	Suppresses bone weakening	(142)
Secondary Bile Acids	Conversion by gut microbiota of primary bile acids	Bind to nuclear receptors, modulating immune responses	Role in maintaining bone mineral density	(143)
Sphingolipids	Cellular metabolism (not microbiota derived)	Bioactive lipids participating in cellular signaling, specifically S1P signaling	Regulate osteoclast differentiation and bone remodeling	(144)
Indolepropionic Acid (IPA)	Tryptophan degradation by gut bacteria (*Clostridium* sp*orogenes*)	Antioxidant activity and gut barrier modulator	Protects bone architecture by reducing oxidative stress	(145)

**Figure 1:**
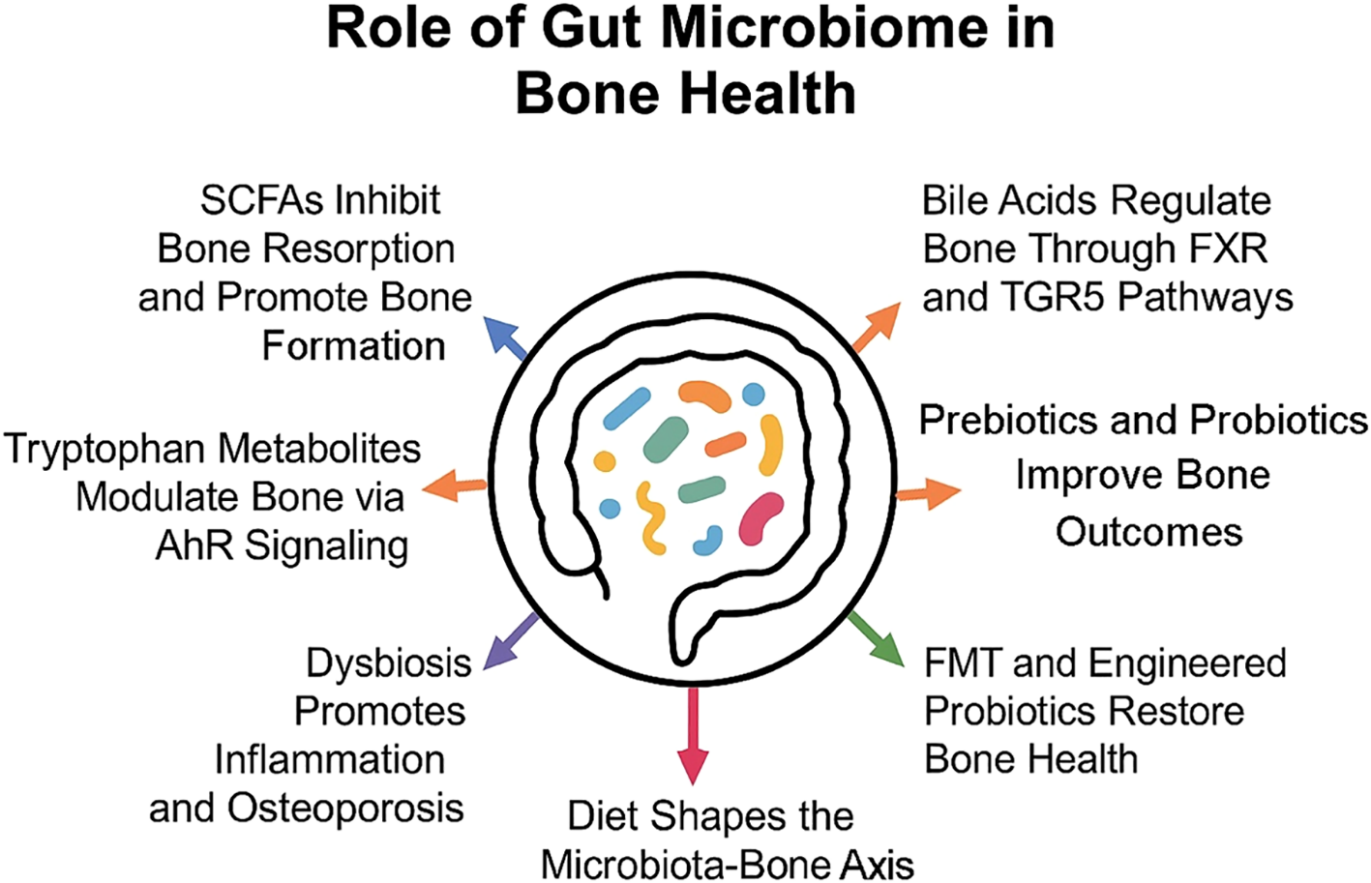
Gut microbiota influences bone health through metabolites and immune modulation, with diet and therapies like probiotics and FMT shaping these effects.

During the past decade, the gut microbiome has emerged as one of the most favorable therapeutic targets for interventions to improve bone health and prevent osteoporosis. More research discovers the intricate association between gut microbiota and bone metabolism. Diet-based interventions, as well as probiotics and prebiotics, proved to regulate the gut microbiome in ways impacting bone density and skeletal health. Fiber, in conjunction with calcium and probiotics, such as *Lactobacillus* and *Bifidobacterium*, represent some of the dietary components that can positively influence mineral absorption through the reduction of inflammation (90). Prebiotics, such as primarily inulin-type fructans, modulate calcium absorption and stimulate the proliferation of beneficial bacteria that produce SCFAs (79). Inulin-type fructans from sources such as chicory root and Jerusalem artichokes are fermented by gut microbes into SCFAs, which provide beneficial effects on bone by increasing calcium absorption due to a lower gut pH. This supports conditions that favor increased BMD and strength (91). It is reported that butyrate and propionate have an inhibiting effect on osteoclast differentiation and bone resorption. On the other hand, they stimulate calcium absorption through the colon. Specific bacterial groups known to be SCFA producers have now become targets to harness the gut microbiome for skeletal health (92). **Figure 1** summarizes the key ways in which gut microbiota and its metabolites influence bone health.

## 5 Diet, microbiota, and bone: a tripartite alliance

Higher-protein, animal-based diets can shift the gut microbiota composition, as they increase bacteria like *Bacteroides*, *Alistipes*, and *Bilophilia*, and also reduce beneficial microbes such as *Lactobacillus* and *Roseburia* (93). Plant-based sources of lipids, nuts, and vegetable oils seemed to favor this gut microbiota diversity (94). In humans, long-term dietary habits are considered to have the maximum impact on microbiota diversity in an individual during neonatal life and thereafter. SCFAs produced within the microbiome reduce the gut pH and have systemic effects that may indirectly support bone health (95). Indeed, the balance between healthy and harmful metabolites is determined by the type of diet that one consumes. Studies showed that diets high in fiber elevate carbohydrate-active enzymes (CAZymes) in the gut and thus reduce inflammation-related proteins (18). Relatively stable and long-term dietary intake is a central determinant for establishing the gut microbiota. However, short-term dietary changes can rapidly alter microbiota, and some enterotypes remain stable over time (93). Long-term dietary interventions seem to modulate bone metabolism, consequently through changes in the gut microbiota (6). **Table 2** summarizes key bacterial genera and their roles in bone health. Indeed, the quick changes in gut microbiota owing to alteration in diet could provide an opening for developing dietary interventions against bone disease. For example, calcium supplementation supports bone health and may indirectly influence gut microbiota and inflammation, which may unlock the complex dietary interactions with gut microbiome metabolism and bone health (95). Moving forward, more comprehensive and integrated research is warranted to know the full impact of such processes on bone health and the treatment of bone disease (92).

**Table 2:** Summary of key bacterial genera and their Roles in bone health (Section 4).

Bacterial genus	Relevant metabolite	Key roles	Impact on bone health	Associated diet/intervention	References
*Lactobacillus*	SCFAs (butyrate, acetate)	Modulate immune responses and reduce inflammation	Inhibit osteoclast activity and promote bone formation	Fiber-rich diet, probiotic supplements	(76, 146)
*Bifidobacterium*	SCFAs (butyrate, propionate)	Enhancement of calcium absorption gut barrier integrity	Increase BMD, decrease inflammation	Prebiotic-rich diet; inulin supplementation	(100)
*Bacteroides*	Bile acids	Influence FXR and TGR5 signaling	Regulate calcium absorption and increase bone remodeling	High-fiber diet, bile acid related therapies	(133, 135, 147)
*Clostridium*	Tryptophan metabolites	Activate AhR and modulate immune responses	Reduced bone resorption and increased osteoblast differentiation	High protein diet, tryptophan supplementation	(86)
*Roseburia*	SCFAs	Maintenance of gut barrier function and anti-inflammation	Supports bone mass and skeletal health	Diet rich in resistant starch and fiber	(148, 149)
*Faecalibacterium*	Butyrate	Anti-inflammatory effects and improve gut homeostasis	Maintain bone integrity and prevent bone loss	Prebiotics, Mediterranean diet	(100, 148, 150)
*Prevotella*	Bile acids	Metabolize bile acids; modulate FXR and TGR5 signaling	Maintain bone mineralization; regulate activity of osteoblasts	High-fiber diet, low-fat diet	(133, 135, 147)
*Akkermansia*	Propionate	Improve gut barrier and reduce inflammation	Enhance bone strength and promote mineral absorption	Polyphenol-rich diet	(148, 151)
*Escherichia coli*	Pathogenic metabolites	Produces toxins that cause disease, and impairs gut barrier function	Potentially harmful, as it increases bone resorption under dysbiosis conditions	None. Has been associated with Western diets	(8, 152)

A diet rich in fruit, vegetables, grain, fish, nuts, and low-fat dairy is generally associated with improved BMD (79). The best way to illustrate the effects of diet on bones is by comparing Western and Mediterranean diets. Long-term intake of Western diets has been shown to lead to an increased risk for bone injuries, specifically hip fractures (96). With a balanced proportion of Bacteroidetes and *Firmicutes*, the Mediterranean diet helps reduce inflammation. Nutritional intake in the traditional Mediterranean diet is associated with a proper balance of calcium, vitamin D, and protein, which can account for the low rate of osteoporosis in Mediterranean Basin countries (97). However, a Western diet directly increases the risk of fracture and bone resorption and generally leads to osteoporosis (98). Studies have shown that polyphenols used as a prebiotic substrate can stimulate the development of *Bifidobacteriaceae* and *Lactobacillaceae* and potentially inhibit the growth of *Escherichia coli*, *Clostridium* perfringens, and *Helicobacter pylori* (99). A good diet supplemented adequately with metabolites is also related to a reduction in inflammatory processes and adequate provision of calcium and magnesium. Finally, modulation of gut microbiota may centrally regulate inflammation. In addition, a leaky gut may be associated with systemic inflammation and osteoporosis; therefore, protection from a leaky gut also ensures a wholesome state of the bone (6). Due to dietary intake and probiotics supplementation, healthy gut microbiota suppression of pro-inflammatory cytokines protects against osteoporosis (2).

Prebiotics modulate the gut microbiota and intestinal function, enormously impacting the gut and bone health (100). Prebiotics trigger some helpful bacteria such as Bifidobacteria and Lactobacilli; their fermentation products are mainly lactic acids which can push away the attack by pathogenic microflora (101). Some studies have shown that gut-derived butyrate led to an expansion of the bone marrow’s regulatory T cells and, thus, participated in the upregulation of bone anabolism (102). Other studies indicate that prebiotic action helps bone strength, bone formation, or even osteoblast/clast activity (103). McCabe et al. showed that pre-/probiotic regulation on bone health and calcium levels in mice (103). In chickens, inulin-induced treatment exhibited increased bone mineralization (104). Several randomized clinical trials present direct evidence of associations between dietary modification and variation in gut microbiome composition and structure. The results showed a significant change in the amount of acetate, increased phyla diversity, an abundance of *Firmicutes* and *Bacteroides*, and reduced *Actinobacteria* (105). This apparently indicated that massive nutrition had a robust effect on the gut microbiota. Changes in metabolic profile support a potential benefit of inulin enriched diet on bone health (106). Notably, So et al. carried out a meta-analysis to investigate the intervention of dietary fiber on gut microbiota. They reported an intervention with fructans and galacto-oligosaccharides that resulted in a significantly higher level of fecal *Bifidobacterium* and *Lactobacillus* in their report (101). The increase can be related to the intestine-mineral absorption-enhancing and short-chain fatty acid anti-inflammatory activities (102). These studies have, more importantly, identified a role for dietary ingredients in gut microbiota health for nutritional guidelines concerning bone health.

## 6 Beyond probiotics: innovating therapeutic strategies

Previous studies evaluated traditionally made doenjang (fermented Korean food), a synbiotic food supplemented with Bacillus subtilis and other beneficial bacteria, in an ovariectomized rat model for its efficacy (107). Rats fed with doenjang for a certain period showed conserved BMD and displayed distinct reduced bone resorption markers compared to the control rat group (108). Another study also found that in humans, a probiotic combination of three *Lactobacillus* strains significantly reduced lumbar spine bone loss, especially in early postmenopausal women (109). In the same study, however, the combination, composed of *Lactobacillus* paracasei (DSM 13434) and two *Lactobacillus* plantarum strains (DSM 15312 and DSM 15313), did not prevent further bone deterioration compared to placebo. Moreover, it was found that certain probiotic strains work only in specific health outcomes, showing rather limited effects (110). Notably, one of the recent studies showed that multistrain probiotics improved ulcerative colitis while uncovering a strong need for site-specific probiotic therapies (111). It was also found that the role of probiotics in inflammatory bowel disease (IBD) is unexpectedly strain-specific. Indeed, when the probiotic mixture was evaluated for its effects on antibiotic-associated diarrhea and gastrointestinal symptoms at different escalating doses, the incidence and severity of antibiotic-associated diarrhea were dose-dependent, with the lowest incidence observed in the highest dose group (110). While this points to a promising therapeutic intervention for bone health, the application of probiotics faces challenges related to strain specificity, viability, and colonization efficiency (112). **Table 3** compares therapeutic strategies targeting the gut microbiome.

**Table 3:** Comparison of therapeutic strategies targeting the gut microbiome (Section 5).

Therapeutic strategy	Description	Advantages	Challenges	References
Probiotics	Living bacteria to improve gut microbial composition	Safe, easily available, strengthens immune system	Strain-specific effects, viability, colonization efficacy	(153–155)
Prebiotics	Indigestible fibers with growth of favorable bacteria	Increase production of SCFA and diminish inflammation	Dose-dependent, with individual variability	(104, 156, 157)
Fecal Microbiota Transplantation (FMT)	Donor microbiota are transferred to the recipient	Restores microbial diversity, and this may be beneficial as a possible approach against bone loss due to aging	Having some infection risks; also variability between donors, and no standardization of procedures.	(121, 158)
Engineered probiotics	Genetically modified bacteria that target specific metabolic pathways	Tailored to cause specific health effects, including bone health	Bioethical considerations, horizontal gene transfer risks, and regulation	(159, 160)
Diet-based interventions	Personalized high-fiber, prebiotic, and probiotic diets	Relatively simple to implement and with large microbial diversity reach and SCFA production	Require long-term adherence; variability among individuals in response	(101, 157, 161)

Synthetic biology holds great promise in therapeutic applications based on genetically modified bacteria to achieve beneficial activities, including targeted production of desired metabolites, modulation of the immune system, and reduction of inflammatory markers (113, 114). If probiotics could be engineered by synthetic biology, they may prove more functional and potent in targeted delivery. In recent years, synthetic biology has been extensively used to create genetically modified bacteria for a broad spectrum of therapeutic applications. For example, exopolysaccharide (EPS) produced by the recombinant *Bifidobacterium* longum 35624 has been shown to inhibit osteoclast formation and thus increase bone formation *in vitro* experiments (115). Moreover, orally administered B. longum 35624 could slow down bone loss in an ovariectomized mouse model. However, although the engineered L. plantarum may target bone health, the genes introduced as the result of engineering could influence other gut bacteria in unintended ways (116). For example, if antibiotic resistance genes are passed over to gut bacteria through horizontal gene transfer, this could dangerously increase the chance of the emergence of new multidrug resistance strains (117). Such situations would add antibiotic resistance to the emerging multidrug-resistant bacteria and hence make it difficult to eradicate using the currently available antibiotics (118). Indeed, synthetic biology is a powerful technology that holds great promise in tissue engineering and biomedicine, especially when alluding to the possibility of developing bespoke therapeutic approaches to bone health using engineered probiotics. Realizing this potential requires carefully balanced methods that address related safety and ethical issues. Particular attention should be paid to collateral horizontal gene transfer in food bacteria and the potential induction of antibiotic resistance (119). Bioethical guidelines on synthetic biology regarding the development of technologies that could limit our fight against dangerous pathogens should be properly established and accrue to everybody transparently with public participation and fair access. Discussion on critical concerns and heightened views among participating scientists, ethicists, stockholders of policies, and the public needs to keep going. This collaboration will ensure the development and implementation of probiotics in a responsible and safe manner and reduce their risks (120).

Initially developed for gastrointestinal disorders, FMT is being explored for systemic diseases, including potential impacts on bone health. FMT is a method of introducing healthy fecal material into a recipient to restore a balanced and healthy gut microbiota ecosystem. Recent studies suggest that FMT could be useful to manage bone health disorders. The female rats in an aging model, when obesity was established with accumulated gut alterations, received FMT from young female rats. The control group had no therapy and was analyzed for the small intestines, bone samples, and gut bacteria at weeks 12 and 24 after the intervention by intragastric FMT. At 24 weeks, the bone quantity in terms of volume, fraction, and thickness across the FMT group was noticeably higher than that across the control group (121). These findings indicate that improvements in gut microbiome composition and gut barrier function could be effective means to ameliorate age-related bone loss. Such promising results suggest that FMT could become one of the ways to fight age-related osteoporosis in the future. Separately, the outcomes in formula-fed piglets have shown restored gut health through scaling up the beneficial use of gut bacteria from FMT; such normalization of gut biology and barrier function has suppressed inflammatory responses (122). These findings also emphasize the impact of early-life microbiota modification on systemic health, including bone homeostasis.

FMT influences bone health through complex interactions involving the gut microbiome, immune system, and bone metabolism. Recently, one publication further showed that FMT reconstituted the bone mass in osteoporotic mice, interpreted to relate to changes in gut microbiota composition and metabolic activity (123). A number of its metabolites seem to act directly on the bone cells, while others do by modulating the host immune response to bone resorption and formation (124). Similarly, FMT-mediated changes in the gut microbiome will have an indirect influence on bone metabolism through the regulation of the immune system (125). The gut microbiome is considered to play an important basic role in modulating immune responses and systemic inflammation at levels and along pathways with consequences for bone health (126). Inflammatory processes are involved in bone conditions such as osteoporosis in many ways. A recent review underlined factors in inflammation, namely the modality of action of cytokines and inflammatory osteoclasts (127). In this process of inflammation, a disturbed balance in the balance between the formation and resorption of bone, therefore, predisposes them to frail bones and osteoporosis. As a treatment technique targeting the gut microbiome, one would expect both systemic and local effects of FMT on inflammation, which could be rather important in modulating bone health (128).

A surprising connection, which is being identified between the gut microbiome and health, is becoming more and more linked to the results coming from microbial metabolism studies. This has thus led to therapy that incorporates the direct supplementation of metabolites implicated in bone health, particularly microbial metabolites. As SCFAs and other microbial metabolites present enormous promise for bone health, several challenges must be addressed in order to allow the current potential to manifest in practical life (95). There may be many differences in the individual responses to metabolite supplementation. The crucial aspect is being aware of and recognizing these complexities in order to adjust appropriate treatment options. A proper understanding of the metabolite effects on bone metabolism will thus be central to the development of targeted therapies, such as possible interactions between metabolites, gut microbiota, and bone cells or even more systemic effects of metabolite supplementation across the body (8).

Unlike traditional pharmaceuticals, microbiome-based therapeutics entail living organisms or complex microbial groups, making the regulatory classification quite a challenge (129). This complexity is further increased by the development of standardized manufacturing processes. The establishment of product stability and consistency are critical approval requirements. Variables like diet and lifestyle should be taken into consideration when designing clinical trials for new microbiome-based therapies, which also involve the need for proper control groups and robust analysis methods for acquired data (130). An important obstacle in undertaking such studies is to choose appropriate endpoints and suitable biomarkers. These will better show the therapeutic effects on both the microbiome and the human host. Other variables include the intrinsic variability in the composition of microbiomes among people. Importantly, individual human microbiome varies depending on diet, lifestyle, and antibiotic history (130). These are key variables that need to be controlled and considered for designing clinical trials. Thus, innovative designs of trials and analytic methods would be critical as both the potential therapeutic impacts and their associated risks are rising. New approaches are needed to extract the effects of microbiome-based interventions from the compounding variables (129). Notably, the regulatory and clinical trial landscape for therapeutics developed from the microbiome changes daily, calling for regulatory bodies to work with scientific experts to address these challenges and establish specific guidelines to evaluate therapeutic microorganisms. Precise guidelines, superior analytics, and innovative collaboration between regulators and scientists would establish the continued exploration of microbiome-based therapeutics, enabling these therapeutics as safe and effective treatments for a wide range of bone conditions.

## 7 Conclusion

The gut microbiota has become a significant determinant of bone health, directly modulating skeletal metabolism through intricate biochemical and immune-regulating mechanisms. Gut microbiota modulation could be an innovative strategy with potential as a treatment option for osteoporosis and other bone disorders. Interventional methods like probiotics, prebiotics, fecal microbiota transplantation (FMT), and synthetic biology are highly prospective measures to improve bone mineral density (BMD) and reduce fracture risk by modulating inflammatory mechanisms, enhancing calcium absorption, and inhibiting osteoclast activity. There is evidence from preclinical and clinical trials that probiotic strains such as *Lactobacillus* and *Bifidobacterium* may favorably modulate bone turnover, whereas SCFAs, bile acids, and microbial metabolite-derived tryptophan are able to suppress bone resorption and stimulate osteogenesis. Furthermore, innovative methods such as engineered probiotics and microbiome-specific dietary interventions hold promise as new horizons for individualized therapies. However, to transfer these advances into clinical application, these challenges must first be tackled: individual microbiomes are highly variable, long-term efficacy of interventions remains unproven, and there are issues of regulation over microbiome-based therapies. Future investigation needs to aim to optimize strain-specific effects, improve colonization efficiency, and establish standardized protocols in order to maximize therapeutic benefit. Unlocking the potential of the gut microbiome to manage osteoporosis not only presents a new avenue of therapy but also lays the ground for precision bone health medicine. Combining microbiome science with bone metabolism can transform the prevention and treatment of osteoporosis and hold the promise of more personalized and effective methods in skeletal care.
